# Polycystic Ovarian Syndrome: Prevalence, Predisposing Factors, and Awareness Among Adolescent and Young Girls of South India

**DOI:** 10.7759/cureus.27943

**Published:** 2022-08-12

**Authors:** Ayesha Jabeen, Veepuri Yamini, Amtul Rahman Amberina, Mummareddi Dinesh Eshwar, Sabitha Vadakedath, Gulam Saidunnisa Begum, Venkataramana Kandi

**Affiliations:** 1 Biochemistry, Mahavir Institute of Medical Sciences, Hyderabad, IND; 2 Biochemistry, Prathima Institute of Medical Sciences, Karimnagar, IND; 3 Biochemistry, College of Medicine and Health Sciences, Sohar Campus, OMN; 4 Clinical Microbiology, Prathima Institute of Medical Sciences, Karimnagar, IND

**Keywords:** awareness, prevalence, body mass index (bmi), endocrine, girls, young and adolescent, polycystic ovarian syndrome (pcos)

## Abstract

Introduction

Polycystic ovarian syndrome (PCOS) is a leading cause of infertility among women throughout the world. PCOS is an endocrine abnormality that presents as hyperandrogenemia, anovulation, and/or polycystic ovaries. The exact causes of PCOS are not entirely understood. However, PCOS may be hereditary and associated with abnormalities such as high body mass index (BMI) and obesity, among others. This study is carried out to assess the prevalence and awareness among adolescents and young girls belonging to South India.

Methods

This was a prospective study that included 250 adolescent and young girls aged between 13 and 25 years. Demographic details including age, educational status, and family income were collected from all the participants after obtaining informed consent. BMI was measured in all the participants. A pre-test was conducted to assess the level of awareness followed by a 30-minute informative briefing on PCOS. All participants were asked to fill up a post-briefing questionnaire. The data collected were processed using statistical software SPSS 11.0, and the chi-square test was applied to find out the significance of the results.

Results

Of the total 250 participants included, the mean age was 16.96 years and most participants (78%) belonged to the age group of 13 to 19 years. Most (78%) of the study participants had normal BMI (18-24.9 kg/m^2^), 17.6% were underweight (BMI < 18), and 4.4% were overweight (BMI > 25). A PCOS prevalence rate of 6.8% was noted among the study participants. A majority (78.4%) of the study participants were unaware of PCOS, and 6.8% were being treated for PCOS. The source of knowledge of PCOS was majorly teachers (37%), followed by doctors (31.5%), the internet (11%), and friends (7.5%). Lack of information and publicity (63%) were found to be the most significant reason for low levels of awareness.

Conclusion

PCOS is a common health problem among adolescents and young girls. Most study participants were unaware of the symptoms and management of the condition. Therefore, the disease burden noted in this study does not necessarily depict the real prevalence. Increasing awareness programs will facilitate improved understanding, increased diagnoses, and effective management of PCOS.

## Introduction

Polycystic ovarian syndrome (PCOS) is a common endocrine/hormonal disorder that affects women, especially after puberty. Alternatively, PCOS may be expanded as polycystic ovary syndrome. Around 5-15% of women in the reproductive age group suffer from hormonal imbalances that lead to menstrual irregularities, cysts in ovaries, infertility, and other health problems that include cardiovascular complications, type 2 diabetes mellitus (T2DM), and endometrial cancer [1]. PCOS is recognized as an important reproductive as well as a metabolic disorder since it affects the ovaries, and 40% of the affected women suffer from insulin resistance and are subsequently predisposed to developing T2DM [2]. The global prevalence of PCOS is estimated between 4% and 20% [3].

The World Health Organization (WHO) data suggests that approximately 116 million women (3.4%) are affected by PCOS globally [4]. The data on the prevalence of PCOS in India are scarce. According to the National Health Portal of India, the prevalence rate of PCOS in Maharashtra was noted to be 22.5% [5]. Another previous report from South India, which included adolescents, showed an incidence of 9.13% [6]. However, the diagnostic criteria for PCOS were different in those studies.

The reasons for the development of PCOS have not yet been precisely identified. However, some previous studies show that PCOS could be linked to hereditary, lifestyle, and environmental factors that include early age of puberty, premature fetal development, family history of PCOS among first-degree relatives, physical inactivity, stress, and obesity among others [7,8]. It was also identified that the steroidogenic enzyme, cytochrome P450 enzymes (CYPs) related gene polymorphisms (CYP11A1, CYP17A1, and CYP19A1) could contribute to the development of PCOS [7].

Patients suffering from PCOS may present with irregular or anovulatory cycles with signs of hyperandrogenism such as acne, seborrhea, hirsutism, alopecia, and polycystic ovaries on pelvic sonography. Other prominent features that PCOS patients show include weight gain and mood swings [6]. PCOS among adolescents and young girls is generally undiagnosed and underreported. Moreover, untreated patients develop complications such as infertility, pregnancy-related issues, metabolic disorders, and cardiovascular diseases [9,10]. PCOS patients have an increased chance of developing impaired glucose tolerance and suffer from T2DM. Also, an association of PCOS with gynecological cancers should be considered a cause for serious concern [11].

The diagnosis of PCOS can be performed by using various guidelines/criteria as recommended by the National Institute of Health (NIH), Rotterdam criteria, and the Androgen Excess PCOS Society (AE-PCOS) criteria [12]. Recently, the international evidence-based guidelines suggested that the Rotterdam criteria are superior to others in diagnosing PCOS [13]. Due to a lack of awareness, many menstrual problems including PCOS remain unidentified and untreated, especially among the rural population. Such patients fail to conceive and present themselves at fertility clinics. Therefore, it is increasingly important to improve awareness among females at an early age, which potentially contributes to early diagnosis and prevents late sequelae of PCOS [14].

This prospective study was carried out to assess the prevalence and knowledge of PCOS among adolescents and young girls. An informative briefing on PCOS was used to improve the basic knowledge of PCOS, and a questionnaire-based survey (pre- and post-test) was carried out to identify the level of knowledge, barriers to awareness, complications, and potential predisposing factors for the development of PCOS.

## Materials and methods

A prospective study was conducted between July and August 2019 and included 250 young and adolescent girls attending schools and colleges in and around Vikarabad, Telangana, India. All the participants of the study were explained the procedure and informed consent was obtained. The study was approved by the Institutional Ethics Committee of Mahavir Institute of Medical Sciences, Telangana, India. Convenient sampling was applied to recruit adolescent and young girls aged 13-19 years and 20-25 years, respectively. Females aged less than 13 years and more than 25 years were excluded from the study.

Data collection procedure

The data were collected in the form of pre-validated questionnaires designated as pre- and post-test questionnaires. The pre-test questionnaire was applied to collect data including sociodemographic details, information regarding awareness of PCOS, causes or predisposing factors, symptoms, complications, and measures to be taken to prevent PCOS. Thirty-minute chalk and board teaching methodology was used along with some charts of the reproductive system to explain the keynotes and provide information regarding PCOS. All the study participants actively participated in the session, which was interactive, wherein questions and doubts raised by the participants were addressed.

A post-test questionnaire was used to assess the perception of students regarding the effect of awareness programs on students' satisfaction and knowledge, reasons for barriers to the lack of awareness, and suggestions for increasing awareness.

Statistical analysis

The data collected were systematically entered into a Microsoft Office 2019 Excel sheet (Microsoft® Corp., Redmond, WA), and were used to prepare tables and calculate the mean and percentage. The data collected were processed using statistical software SPSS 11.0 (IBM Corp., Armonk, NY), and the chi-square test was applied to find out the significance of the results.

## Results

The mean age of the study participants was 16.95 years. Among them, the majority were adolescents (78.8%) followed by young girls (53%). Of the participants included in the study, 35.6% were high school girls, 16% were junior college girls, 28.4% were non-medical undergraduates, and 20% are medical undergraduates. The BMI was found normal in 78% of study participants, 17.6% were underweight, and 4.4% were noted to be overweight. The sociodemographic details and other aspects of the study participants are shown in Table 1.

**Table 1:** Demographic data, body mass index, and knowledge of PCOS among study participants BMI, body mass index; PCOS, polycystic ovarian syndrome

Parameter	Variable	Number of participants (n=250) (%)
Age group	Adolescents (13-19)	197 (78.8)
	Young girls (20-25)	53 (21.2)
Educational level	High school	89 (35.6)
	Junior college	40 (16)
	Non-medical graduates	71 (28.4)
	Medical graduates	50 (20)
Family income (per month in dollars)	<75	23 (9.2)
	76-149	54 (21.6)
	>150	173 (69.2)
BMI (kg/m^2^)	<18 (underweight)	44 (17.6)
	18-24.9 (normal)	195 (78)
	>25 (overweight)	11 (4.4)
Knowledge of PCOS	Adolescents	9 (3.6)
	Young girls	45 (18)
Being treated for PCOS	Yes	17 (6.8)
	No	233 (93.2)

Only 54 (21.6%) study participants were aware of the term PCOS, and 6.8% had already been diagnosed and were receiving treatment for PCOS. Among the study participants who were aware of PCOS, the majority acquired information through teachers (37%), followed by doctors (31.5%), the internet (11.1%), and other sources (12.9%), and a few participants collected information from friends (7.5%). The awareness of PCOS was more among young women (84.9%) as compared to adolescent girls (4.5%). Similarly, participants who were pursuing undergraduate (both medical and non-medical) degrees had higher awareness levels (92%) as compared to the pre-university girls (2.5%). A pre- and post-test comparison of awareness among the study participants regarding the potential reasons for developing PCOS is delineated in Table 2.

**Table 2:** Comparison of awareness among the study participants regarding causes of polycystic ovarian syndrome *Statistically significant

Variable	Choice	Pre-test (n)	Post-test (n)	p-Value
Genetic	Know	45	218	<0.0001*
	Don’t know	205	32	
Obesity/overweight	Know	43	202	<0.0001*
	Don’t know	207	48	
Hormonal Imbalance	Know	54	215	<0.0001*
	Don’t know	196	35	

The awareness levels of the study participants regarding the symptoms of PCOS along with the pre- and post-test comparisons are delineated in Table 3.

**Table 3:** Comparison of awareness of symptoms of polycystic ovarian syndrome among the study participants *Statistically significant

Variable	Choice	Pre-test (n)	Post-test (n)	p-Value
Irregular menses	Know	89	228	<0.0001*
	Don’t know	161	22	
Hirsutism	Know	44	212	<0.0001*
	Don’t know	206	38	
Acne	Know	60	217	<0.0001*
	Don’t know	190	33	
Mood swings	Know	70	221	<0.0001*
	Don’t know	180	29	
Weight gain	Know	52	211	<0.0001*
	Don’t know	198	39	
Hair loss	Know	46	224	<0.0001*
	Don’t know	204	26	
Hyperpigmentation	Know	48	207	<0.0001*
	Don’t know	202	43	

The awareness levels of the study participants regarding the complications of PCOS along with the pre- and post-test comparisons are delineated in Table 4.

**Table 4:** Comparison of awareness of complications of polycystic ovarian syndrome among the study participants *Statistically significant

Variable	Choice	Pre-test (n)	Post-test (n)	p-Value
Diabetes	Know	33	210	<0.0001*
	Don’t know	217	40	
Cardiovascular disorders	Know	21	207	<0.0001*
	Don’t know	229	43	
Infertility	Know	81	211	<0.0001*
	Don’t know	169	39	
Abortion	Know	72	224	<0.0001*
	Don’t know	178	26	
Breast cancer	Know	43	215	<0.0001*
	Don’t know	207	35	

The awareness levels of the study participants regarding the measures required to prevent PCOS along with the pre- and post-test comparisons are delineated in Table 5.

**Table 5:** Comparison of awareness of measures required to prevent polycystic ovarian syndrome among the study participants *Statistically significant

Variable	Choice	Pre-test (n)	Post-test (n)	p-Value
Healthy diet	Know	176	242	<0.0001*
	Don’t know	74	8	
Weight control	Know	117	239	<0.0001*
	Don’t know	133	11	
Physical activity	Know	152	243	<0.0001*
	Don’t know	98	7	
Reduce stress	Know	100	240	<0.0001*
	Don’t know	150	10	
Avoid smoking and alcohol	Know	111	228	<0.0001*
	Don’t know	139	22	

The reasons for the lack of knowledge and the barriers to information regarding PCOS included lack of information (45.2%), no advertisements (18%), and social stigma (5.6%), among others, as shown in Table 6.

**Table 6:** Barriers responsible for the lack of awareness of polycystic ovarian syndrome among the study participants

Barrier	Study participants, n (%)
Lack of information	113 (45.2)
Lack of advertisements	45 (18)
Social stigma	14 (5.6)
Improper knowledge	13 (5.2)
Illiteracy	6 (2.4)

The study participants suggested that conducting surveys (28%), awareness programs (22%), campaigns (4.4%), advertisements (3.6%), and seminars (2%) could improve the knowledge and awareness of PCOS among the susceptible populations.

## Discussion

PCOS is one of the major endocrine diseases of concern among women. PCOS results in hormonal, gynecological, metabolic, and cosmetic disorders. It was first described by Stein and Leventhal in 1935 among women, and the prevalence ranges between 5% and 15% depending on the criteria applied to diagnosis [1].

According to the International Classification of Diseases (ICD), PCOS is a complex disease that manifests as infertility, hirsutism, obesity, and menstrual disturbances such as oligomenorrhea, amenorrhea, and anovulation. PCOS is associated with bilateral enlarged ovaries studded with atretic follicles and evidence of fluid-filled cysts as identified by ultrasound scanning [15].

Mechanisms involved in the development of PCOS and associated complications

The mechanisms underlying the development of PCOS and complications associated with it appear to be influenced by several factors. Elevated activities of testosterone were found among the patients suffering from PCOS. This may contribute to the reduced activities of follicle-stimulating hormone (FSH) and disrupt the function of ovaries. Hyperandrogenism also causes hirsutism, elevated insulin secretion, glucose intolerance, dyslipidemia, and T2DM. The hormonal imbalances occur due to the disturbed hypothalamic-pituitary-ovary axis and result in menstrual irregularities, anovulation, and infertility. Most patients who developed PCOS had a genetic predisposition [7]. Moreover, environmental factors, food, and lifestyle were influential in the development of PCOS and associated complications [3].

Currently, PCOS has no definite cure, but the associated comorbidities can be addressed to improve the quality of life and minimize the long-term complications associated with PCOS. The metabolic disturbances worsen with time, and the prognosis becomes poor gradually over time. The time lag between the appearance of symptoms and diagnosis of PCOS needs to be lessened to reduce the deleterious effects that include infertility, impaired glucose tolerance, insulin resistance, obesity, cardiovascular complications, and endometrial cancer, among others. Moreover, PCOS also affects the health of post-menopausal women, as evidenced by the increased occurrence of obesity and T2DM among postmenopausal women with PCOS as compared to others [11,16].

A timely diagnosis of PCOS in symptomatic adolescent girls is important for the initiation of appropriate treatment and management initiatives. However, this can be achieved by spreading awareness through educational interventions and other measures such as educating the susceptible population regarding the symptoms, etiology, age of onset, and PCOS-related health care services [14].

The present study included the majority of adolescents, and the awareness among them was critically poor when compared to the young girls in the age group of 20-25 years who had a better knowledge of PCOS. This probably might be due to less exposure to society, i.e., the parents of the participants belonging to rural backgrounds restrict their social behavior. This, in a long run, could have created stigma regarding the expression of their enthusiasm toward the physical and mental transformations occurring after menarche.

In our study, the participants were briefed on the facts of PCOS, and an effort was made to explain them logically. Enlightening the age group of 13-19 years was found to be more fruitful compared to the young girls, probably due to rigid perceptions in the latter group.

A 21.6% awareness was observed in the current study, which was on the lower side in contrast to the results obtained in a study from Indore, central India, as reported by Patel and Rai who reported an awareness level of 41% [17]. This suggests that the women in the age group of >18 years, who are studying in colleges, and working women might have acquired knowledge regarding PCOS through socializing, news, media, workshops, conferences, etc. Another study by Rajkumari et al. from Orissa, East of India, reported 22% awareness, a similar finding to our study results [18]. The reason could have been the inclusion of rural and semi-urban populations. A study conducted among non-medical undergraduates in Dhaka, Bangladesh, revealed that more than 50% of the participants had knowledge of PCOS, which was significantly higher than our result [19]. This might be due to the rural background of our participants who had restricted access to information.

Most study participants were unaware of the symptoms of PCOS and that this condition may result from genetic reasons and can possibly be inherited from parents and predisposing factors for the development of PCOS including obesity, among others. The majority believed that hormones and their imbalances are related to the development of PCOS and that symptoms such as irregular menses, mood swings, hirsutism, and hair loss could be attributed to PCOS. Most participants were aware that infertility and abortion could be long-term sequelae associated with PCOS. However, they were not aware of the association of PCOS with cardiovascular complications, diabetes, and gynecological cancers. The awareness regarding symptoms and complications of PCOS was significantly increased after the educational intervention done in our study. Knowledge about healthy diet and physical activity/exercise and their beneficial effect on the prevention and management of PCOS was noted among the study participants, a finding similar to that reported in a previous study by Pitchai et al. from Mumbai, India [20].

Teachers and doctors were noted to play a key role in the dissemination of information regarding PCOS. It was found that the level of education positively correlated with the levels of awareness. The highest awareness percentages were noted among medical undergraduates followed by non-medical graduate students.

Limitations

The PCOS prevalence rates observed in this study do not necessarily reflect the real prevalence. A selected age group of participants were enrolled (adolescents and young girls), and other women, of different ages, and those who reached menopause were excluded from the study. Also, the diagnosis of PCOS among the study participants was not performed based on definitive diagnostic criteria. The inclusion of limited subpopulations and subgroups from a defined geographical area could have contributed to bias in the results obtained from this study.

## Conclusions

PCOS is a common problem encountered by females after attaining puberty. Most study participants were unaware of the symptoms and management of the condition. Therefore, the disease burden noted in this study does not necessarily depict the real prevalence. PCOS is an endocrine disease associated with hormonal imbalances wherein women suffer from hyperandrogenism and increased activities of testosterone. PCOS presents as irregular menstruation, anovulation, and/or polycystic ovaries. It affects women of all age groups, and the knowledge of PCOS is essential to efficiently manage the condition and minimize the long-term complications associated with it like insulin resistance, glucose intolerance, diabetes, cardiovascular disease, and endometrial cancers, among others. However, the prevalence of PCOS and the knowledge of symptoms, complications, prevention, and management of this condition are highly variable in different geographical regions and population groups. Owing to the complexity of the disease, its presentations, and potential complications, efforts should be made by the governments to increase its awareness, especially among the rural and low socioeconomic populations.

## Appendices

Study questionnaire:

PRE-TEST

Consent: Are you willing to participate in this survey: yes/no

     Part  1 (sociodemographic information)

Name:                                                    Age:                                                         Residence:   

Height (cm):                                         Weight (kg):                                          BMI(kg/m^2^):

Education level

 Field of study

 Marital status

 Monthly income in rupees

A. High school

A. Health care

A. Unmarried

A. <5,000

B. Junior college

B. Non‑health care

B. Married

B. 5,000‑10,000

C. Undergraduates

C. Divorced

C. >10,000

Tick the appropriate response for the following:

 Part 2

Are you aware of PCOS: YES/NO

 If yes then the source of information:

Are you being treated for PCOS: YES/NO

What type of doctor would you prefer:

A. Teacher

B. Friend

C. Doctor

D. Internet

E. Newspapers

F. Any other source

A. Dermatologist

B. Gynecologist

C. Ayurvedic

D. Homeopathic

v Do you think the below factors can cause PCOS:

s.no

Cause

Yes

No

Don’t know

A.Genetic (family history)

B. Obesity

C. Hormonal imbalances

v Do you know whether the following are the symptoms of PCOS or not:

S.no

Symptom

Yes

 No

 Don’t know

A. Irregular menstrual cycles

B. Excess facial and body hair

C. Acne (pimples)

D. Mood swings

E. Weight gain

F. Hair loss

G. Hyperpigmentation (dark skin patches)

v Are you aware of complications of PCOS:

s.no.

Complication

Yes

No

Don’t know

A. Diabetes

B. CVS disease

C. Infertility

D. Abortion

E. Breast cancer

v Knowledge of measures to be taken to prevent PCOS:

s.no.

Measures

Yes

No

Don’t know

A. Healthy diet intake

B. Weight control

C. Regular physical activity

D. Reduction of  stress levels

E. Avoid smoking and drinking

                                                                    ====×====

POST-TEST

Name:

Part 2

Are you aware of PCOS: YES/NO

 If yes then source of information:

Are you being treated for PCOS: YES/NO

What type of doctor would you prefer:

A. Teacher

B. Friend

C. Doctor

D. Internet

E. News papers

F. Any other source

A. Dermatologist

B. Gynecologist

C. Ayurvedic

D. Homeopathic

v Do you think the below factors can cause PCOS:

s.no

Cause

Yes

 No

Don’t know

A. Genetic (family history)

B. Obesity

C. Hormonal imbalances

v Do you know whether the following are the symptoms of PCOS or not:

S.no

Symptom

Yes

 No

 Don’t know

A. Irregular menstrual cycles

B. Excess facial and body hair

C. Acne(pimples)

D. Mood swings

E. Weight gain

F. Hair loss

G. Hyperpigmentation (dark skin patches)

v Are you aware of complications of PCOS:

s.no.

Complication

Yes

No

Don’t know

A. Diabetes

B. CVS disease

C. Infertility

D. Abortion

E. Breast cancer

v Knowledge of measures to be taken to prevent PCOS:

s.no.

Measures

Yes

No

Don’t know

A. Healthy diet intake

B. Weight control

C. Regular physical activity

D. Reduction of  stress levels

E. Avoid smoking and drinking

Ø Is this survey helpful to you: YES/NO

Ø Did you gain any knowledge related to PCOS through this session : YES/NO

Ø What do you think are the barriers for decreased awareness in your region:

_____________________________________________________________________________________________________________

Ø Do you think the topic of PCOS to be included the academic curriculum in high schools: YES/NO

Ø Any improvements required to increase the awareness of PCOS in general population:

______________________________________________________________________________________________________________

                                                    THANK YOU

Informed consent form:

Title of Project:

I, Mrs. /Miss/Mr. _______________________ give my consent to be part of this study.

I have read and understood the information provided or it has been read and explained to me.

I have had the opportunity to ask questions about the research and all the questions that I have asked have been answered to my satisfaction. I consent to be a participant in this research work.

I also understand that participation in this study is completely voluntary and I have been informed that I can withdraw from the study anytime if I decided not to participate.

Signature of Participant: _____________________________________________________

Age: ___________ Date: ________________ Time: __________________

If participant is unable to give consent

I have read the provided information or it has been read and explained to me. I have had the opportunity to ask questions about the research and all the questions that I have asked have been answered to my satisfaction. I give my permission for participant to take part in this research work.

Name of the Legal Representative: ___________________________________________

Date: ______________ Time: ____________ Signature: _________________________

Statement by researcher/person taking consent

I have correctly read out the information sheet to the participant, and to the best of my ability made sure that the participant understands the information included. I confirm that the participant was given an opportunity to ask questions about the study, and all questions asked by the participant have been answered correctly and to the best of my ability. I confirm that the individual has not been pressurized, into giving consent, and the consent has been given freely and voluntarily.

Name of the researcher: ____________________________________________________

Date: ______________ Time: ____________ Signature: _______________________
